# “Might relapse today” - The categorization of discussions in the r/benzorecovery subreddit

**DOI:** 10.1192/j.eurpsy.2022.2145

**Published:** 2022-09-01

**Authors:** R. Friberg Von Sydow

**Affiliations:** History and Contemporary Studies, Södertörn University, Huddinge, Sweden

**Keywords:** Recovery, Self-help, Reddit, Benzodiazepines

## Abstract

**Introduction:**

The social media platform Reddit is a contemporary context where we have an opportunity to identify problems experienced by people regarding different aspects of life. The platform is virtually anonymous which might make users discuss their problems more freely. Reddit is divided in subreddits where different subjects are discussed and the discussions are controlled by creators and moderators. I have identified a quite active subreddit targeted towards recovering addicts of benzodiazepines; r/benzorecovery.

**Objectives:**

* To analyze strategies of recovery in user narrative * To identify techniques commonly used and the how they are described * To construct metadata in order to assess how frequent the discussion of a different techniques are

**Methods:**

Technically, what is done in this study, is adding mark-up metadata to different discussion. A rudimentary form of analysis suitable with a larger digital corpus where content metadata is added (Gilliland Swetland 2000). The metadata is constructed through a hermeneutical method in which the researcher analyses the subreddit.

**Results:**

Answering question like: Example: DIY-tapering; different ways to limit drug use by using less. 1) how common are discussion of taperings in relation to other subjects? 2) Is tapering commonly discussed together with other subjects and techniques?

**Conclusions:**

Using a method of categorization and metadata mark-up we could gain a good understanding of the problems among recovering benzodiazepine addicts. We will also have the possibility to identify concepts that addicts themselves discuss and relate these to professional concepts thus creating better possibilities of communication between professionals and clients.

**Disclosure:**

No significant relationships.

